# Availability of open data for spatial public health research

**DOI:** 10.3205/000303

**Published:** 2022-03-04

**Authors:** Manuela Peters, Hajo Zeeb

**Affiliations:** 1 Leibniz Institute for Prevention Research and Epidemiology – BIPS, Bremen, Germany; 2 Faculty 11 – Human and Health Sciences, University of Bremen, Germany

**Keywords:** data infrastructure, open data, open government, open geodata, health research

## Abstract

**Background:** Preventive and health-promoting policies can guide (place- and space-specific) factors influencing human health, such as the physical and social environment. Required is data that can lead to a more nuanced decision-making process and identify both existing and future challenges. Along with the rise of new technologies, and thus the multiple opportunities to use and process data, new options have emerged to measure and monitor factors that affect health. Thus, in recent years, several gateways for open data (including governmental and geospatial data) have become available. At present, an increasing number of research institutions as well as (state and private) companies and citizens’ initiatives are providing data. However, there is a lack of overviews covering the range of such offerings regarding health. In particular, for geographically differentiated analyses, there are challenges related to data availability at different spatial levels and the growing number of data providers.

**Objectives:** This paper aims to provide an overview of open data resources available in the context of space and health to date. It also describes the technical and legal conditions for using open data.

**Results:** An up-to-date summary of results including information on relevant data access and terms of use is provided along with a web visualization. All data is available for further use under an open license.

## Zusammenfassung

**Hintergrund:** Präventive und gesundheitsförderliche politische Maßnahmen können (raumspezifische) Einflussfaktoren auf die menschliche Gesundheit, wie z.B. die der physischen und sozialen Umwelt, steuern. Grundlage sind Daten, die zu einer nuancierten Entscheidungsfindung führen und sowohl existierende als auch zukünftige Herausforderungen identifizieren können. Mit der Einführung neuer Technologien und damit der vielfältigen Möglichkeiten zur Nutzung und Weiterverarbeitung von Daten haben sich neue Möglichkeiten ergeben, Faktoren, die sich auf die Gesundheit auswirken, zu messen und monitorieren. So sind in den letzten Jahren Portale für offene Daten (inklusive Verwaltungs- und Geodaten) entstanden. Immer mehr Forschungseinrichtungen sowie (staatliche und private) Unternehmen und Bürgerinitiativen stellen zudem Daten zur Verfügung.

**Ziel der Arbeit/Methoden:** Bislang fehlt es allerdings an Übersichten, die die aktuelle Bandbreite dieser Angebote im Hinblick auf den Gesundheitskontext erfassen. Insbesondere für räumlich differenzierte Betrachtungen ergeben sich Herausforderungen bezüglich der Datenverfügbarkeit auf den verschiedenen räumlichen Ebenen und der zunehmend unübersichtlich werdenden, heterogenen Palette der Anbieter. Dieser Beitrag soll, basierend auf Web- und Datenbankrecherchen, einen Überblick geben, wo bislang offene Daten im Kontext von Raum und Gesundheit angeboten werden und unter welchen technischen und rechtlichen Bedingungen diese verwendet werden können.

**Ergebnisse:** Eine aktuelle Ergebnisübersicht zeigt die stark heterogene Verfügbarkeit relevanter Datenzugänge und unterschiedliche Nutzungsbedingungen relevanter, raumbezogener Public-Health-Daten auf. Die deskriptive Darstellung wird durch eine detaillierte Webvisualisierung unterstützt und steht inklusive aller zugrundeliegenden Daten barrierefrei und unter einer offenen Lizenz allen Interessierten zur Weiternutzung zur Verfügung.

## Introduction

There is a close relationship between place, space and health, with each location posing different conditions and challenges. For instance, it is not only socioeconomic conditions, environmental exposures and the quality and character of the natural and built environment that vary, but also access to and accessibility of health-related resources. According to the ‘Health in All Policies’ approach, building health-promoting living conditions on a local level should be considered in all areas of policy and administration. This has already been initiated with the Ottawa Charter [1]. Simultaneously, the aim is to prevent health impairments through targeted activities within the framework of situational prevention. Specific measures in line with the Public Health Action Cycle require data-based information. Although the Federal Health Reporting (GBE) system, for example, can draw on a range of data sources, the information available varies depending on the geographical unit and the data situation [2]. As a result of digitization, the supply of data, the number of providers and the associated possibilities for data linkage have grown considerably. In particular, *Open Data* (OD) made available by public authorities, private or state-owned companies, research institutions or citizens’ initiatives allows novel approaches and options for use. The term *Open Government Data* (*OGD*) is currently used to describe a growing number of supply channels for administrative data, along with standards for research and provision, and the offering of selected data through company websites and/or general portals. Compared to other countries, however, Germany is still lagging behind when it comes to exploiting this potential for public health research [3]. One of the main causes is the complexity of the supply market [4]. For instance, only a few local authorities and regional federations run OD portals, which are also only partially integrated into umbrella catalogs or interlinked. Finding data, particularly for specific geographic scales and the subject area discussed in this article, therefore remains challenging. In addition, different technical and regulatory constraints hinder the use of the data. Regarding FAIRification (FAIR is an acronym for findability, accessibility, interoperability and reusability [5]), the problems are very similar to those experienced with research data in general.

## Objectives and methodology

This article seeks to provide orientation on currently available data offerings considering space and health on different administrative levels that can be used and disseminated both barrier-free and without any technical and legal restrictions. The selection of relevant explanatory determinants is based on theoretical models. Since the aim of the project is to capture current practice initiatives, and since hardly any scientific research is available for this context so far, availability of relevant data is examined using a simple web search (Google) and complemented by reviewing references on relevant websites as well as in catalogs and data portals. Based on a snapshot of the current situation, data sources are identified and classified according to theoretical dimensions following a definitional introduction to OD and a description of the conceptual framework. For this purpose, existing freely available datasets (e.g. web visualizations), have been adapted and extended to the new topic. This data is provided for further use under an open license. The information may be relevant for politics, planning and science as well, e.g. as a basis for local activities in the field of prevention and epidemiological studies.

## State of research

In Germany, the interdisciplinary discourse on OD (especially on reuse) is still in its infancy stages and mainly driven in the administrative context of OGD, with only a few comprehensive papers [6], [7], [8] or web visualizations [9], [10], [11] available. To date, apart from solely geodata-based reviews [12], [13], [14] and reviews focusing mainly on administrative data sources for public health research [2], [15], [16], [17], there are no existing overviews for the specific context of space-based health research regarding relevant open data sources, their access modes and possible uses.

## Open data

Published data is not necessarily open. It is not open, for example, if rights of use are retained or granted on a case-by-case basis only, or if the data is provided in a format preventing further use. By definition, data is open (in technical and legal terms) in particular if it is machine-readable and structured and can be freely used, reused and distributed by anyone using an open interface for any purpose and without any restrictions, discrimination or fees [18]. Efficient data reuse therefore implies that it is available in good quality, as raw data, ideally including metadata, and with clearly stated rights of use. Licensing conditions are addressed within the framework of *legal openness*. Most public data can only be used in accordance with declared conditions, e.g. allowing commercial use, processing and dissemination. For personal data protected by law, subsequent use is not permitted in most cases. Table 1 (Tab. 1) provides orientation, listing commonly used licenses that allow further processing in the sense of OD, mostly without any restrictions. *Technical openness* relates to data formats ensuring reuse with both proprietary and free software. In recent years, recommendations for the publication of data have been formulated. The OGD principles listed in Table 2 (Tab. 2) have been considered standard ever since.

### Data sources

**Figure 1:**
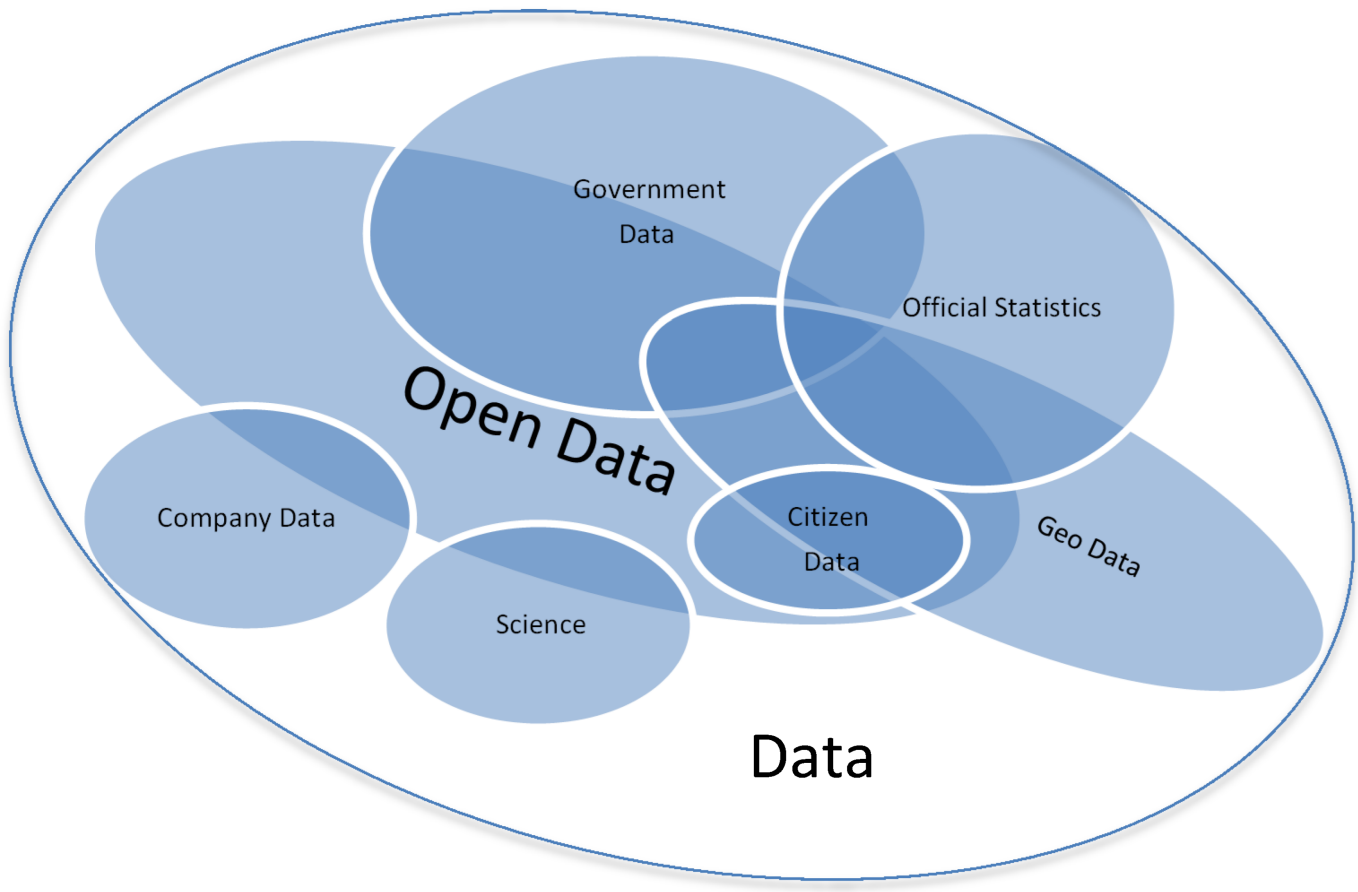
Open data and intersections by data source

In addition to OGD, open data that can be used for spatial public health research includes a wide range of open geospatial data as well as data from official statistics, which in some cases are already linked to geospatial data. Furthermore, companies, research institutions and the civil community offer data in open formats for further use. The sources shown in Figure 1 (Fig. 1) are described in more detail below.

#### OGD and subject data

The term OGD refers to its origin in the public sector (authorities, agencies, state departments) [19]. In many cases, such data is available in an aggregated form only, sometimes it is difficult to find or can only be accessed upon request (e.g. based on the Freedom of Information Act (FOIA)). For instance, numerous authorities provide ordered supplies that are usually free of charge but do not fulfill the criteria of OGD by definition. Whereas OGD has so far been offered mainly on a provider-specific basis, in the future it is to be made available proactively, to anyone, for any purpose, without restrictions and free of charge, so that it can be reused and redistributed [20]. The formats provided and the degree of accessibility determine *whether* or as *how* open OGD can be described [19]. To quantify this and as proof of quality, more and more data providers use the cascading 5-star model proposed by Berners-Lee (the second star can only be awarded to those who already fulfill the requirements regarding the first star). Said model is based on the assumption of an open license and topped by data published in the web in a structured, non-proprietary format with a unique URL to link the data [21]. National OGD platforms such as *Open Data Monitor* or *GDI-DE* have been supported by the federal government for several years, combining regional and municipal offerings. The national metadata platform *GovData.de* does not maintain any data itself but refers to OGD from all federal levels. The Federal Ministry of Transport and Digital Infrastructure (BMVI) hosts multiple access points, including *mdm Portal*, *CODE-DE2*, data from the Copernicus Earth Observation Program and *mCLOUD* (for mobility, geospatial and weather data). The latter also serves as a research platform for OD from private providers in the mobility sector. In addition, Germany’s National Meteorological Service *Deutscher Wetterdienst* (DWD) currently offers free access to open meteorological and climatological data on its *OD portal*. Some ministries do not make data available themselves but through open data portals.

#### Open geodata 

Open geodata emerge from government, research, the private sector or crowd-sourced initiatives such as *OpenStreetMap* (OSM), and can be made available as thematically prepared geospatial data or basic geodata describing properties and topographies [22]. For public health research, the value of this resource arises primarily through *data linkage* with attribute data [23], [24] and hence the ability to identify ecological correlations or spatial disparities in relation to health-related issues [25]. It is necessary that the data is up to date, exhaustive, quality-checked, provided in uniform and interoperable form (e.g. using the harmonized pan-European metadata standard DCDAT), that it has transparent terms of use and documented metadata, and that it is permanently available [26]. By introducing the Infrastructure for Spatial Information in Europe (INSPIRE), the legal basis for this has been established. In Germany, this is implemented through the Infrastructure for Spatial Information in Germany (GDI-DE). Central access points to search for open geodata on all federal levels are provided by *GovData.de* and *Geoportal.de*. OD is also offered by the Federal Agency for Cartography and Geodesy (BKG). Map-compatible datasets are provided in regional databases via interoperable download services. However, these are not necessarily OD in every case. Private companies such as ESRI und Google (*GoogleMaps*) also make selected geodata available for private use using free licenses; however, some of them can only be used with proprietary software. Another resource for health-related spatial data, provided by the National Association of Statutory Health Insurance Funds (GKV-Spitzenverband) and the German Hospital Association (DKG), has been in regular operation since January 2020: The web application *krankenhausstandorte.de* allows providers to register geocoded hospital and outpatient facilities (including metadata), which can then be accessed by users (requires registration).

#### Open data from official statistics

Data from official statistics include geospatial as well as registry, social, environmental and health data. At federal level, the Federal Statistical Office is the leading data provider. It is also possible to obtain official OD from regional databases, local or municipal governments as well as from overarching portals and databases, such as the *INKAR database* of the Federal Institute for Research on Building, Urban Affairs and Spatial Development (BBSR). Social welfare statistics and social reporting also contain relevant OD at federal level and, in some cases, at the small-scale level, available at *state* or *local government gateways*. So far, however, such “atlases of social infrastructure” have mainly been offered in non-machine-readable PDF format.

#### Open company data

The data portal of *Deutsche Bahn AG* is an example of a private company that offers OD on a national scale via its own gateway. Likewise, the *Open Data Platform ÖPNV* initiative, powered by nine major German transport associations, makes OD freely available on its portal. In addition, more and more regional and local transportation services are offering open data on their corporate websites (e.g. *NVBW Open Data*).

#### Open data through Citizen Science (CS)

In addition to government activities, volunteers collect data in crowd-sourced projects and make it available for free (further) use under open licenses. Well-known initiatives include the projects of the OK-Lab Stuttgart with the https://sensor.community/de/ and *OpenSenseMap.org* platforms as well as some projects of the *Codefor-Lab* (https://www.codefor.de/projekte/). The *offenedaten.de portal*, which has, however, been closed in the meantime and on which Germany-wide OD was mapped interactively for the first time on the basis of web-crawler matches, still serves as a prototype for such applications today [13]. Similar concepts are pursued by the *OpenDataAtlas* [9] and *Opendata-City-Census*. The potential of these resources becomes particularly obvious when it comes to aspects such as copyright, terms of use, costs and availability (e.g. of OD missing from the official side or not recorded or offered in a uniform manner).

#### Open research data (Open Science)

To some extent, open research data may also be re-used as well as linked to spatial data. A beneficial development in this context is the growing commitment to *FAIR Data Principles* [5]. However, data meeting these principles does not necessarily have to be open.

(Health) surveys are another potential source of data. To perform regionalization of national surveys, the samples need to be very large, so no usable area-wide, small-scale data is available for many aspects of interest. In some cases, the data is restricted to scientific purposes and therefore available as Scientific Use Files (SUF) only. An international register with detailed information on more than 2,000 research data repositories with open data for re-use, under defined conditions, is provided by the Registry of *Research Data Repositories* platform (*re3data.org*).

## Conceptual framework

Healthy living conditions are a multidimensional concept with complex interactions. Whereas the main focus was on individual [27], [28], [29] and environmental [30], [31], [32] risk factors in the past, today protective determinants in the sense of salutogenesis, such as social structures, availability of environmental resources (e.g. green spaces) and potential factors that may affect physical activity are increasingly taken into account [33], [34]. The holistic human ecology paradigm [35], which identifies influencing factors from social, ecological and economic systems, can be used to organize the data. The dimensions are in line with the aspects that the World Health Organization (WHO) has identified as essential for human well-being based on a review [36]. In addition to individual (lifestyle) factors, these include structural conditions of the living environment, such as (1) sociodemographic and socioeconomic factors, (2) factors of access and reachability, (3) the physical neighborhood, and (4) climate and environmental factors. This theoretical framework serves as the foundation for selecting and organizing the data as presented in the following data research results.

## Results: relevant dimensions and data sources

Although full datasets are available in the field of *environment and health* at national and municipal levels, e.g. from environmental reporting (UBE) and the federal/state indicators of health monitoring, they do not refer to each other [37]. In addition, the data is mainly available in non-open reporting formats. In accordance with the dimensions previously defined, however, the specific data resources focused on below and listed in Attachment 1, Table 3 and the corresponding web visualization (https://sciencemap.github.io/Open-Data-for-Health) can be considered.

### Social-environmental indicators: social condition, sociodemographic and socioeconomic factors

Different levels of exposure and thus health effects are, among other factors, determined by the population’s socioeconomic and sociodemographic composition [38], [39], [40], with social inequality increasing environmental injustice [41]. For epidemiology, the factors education, occupational status and income are defined as the most important characteristics within this dimension [17], [42].

Furthermore, the economic environment, the financial situation of the public sector, and the level of collective wealth reflect the social capital and the capacity of a region to provide services of public interest. A valuable synopsis of data resources along this dimension, including official statistics, welfare statistics and the GBE, has been elaborated by Bardehle et al. [17]. Information on socio-spatial differences in health can be obtained by linking this data with geodata. Moreover, aggregated indicator systems for measuring socioeconomic deprivation have been established for Germany in the past [43]. Of these, the data provided by the German Index of Socioeconomic Deprivation (GISD) [44], for example, are publicly available and presented in machine-readable form; the license, however, allows no further processing.

Well-being can also be affected by personal feelings of vulnerability (e.g. due to crime and traffic safety). Although the Federal Criminal Police Office (BKA) stopped providing its federal-level police crime statistics (PKS) exclusively as PDF files in 2014, small-scale data in machine-readable form is often missing or can only be accessed upon request.

### Availability and accessibility

Access to (care) services relates, among other things, to both general availability and access in the sense of reachability. Social conditions (participation, inclusion) and thus existing social infrastructures are of great importance to health due to their salutogenic protective function [45].

Life expectancy at birth can be interpreted as an indicator of the general living and healthcare conditions in a given region. While family physicians represent a key institution in outpatient healthcare, recent years have witnessed an increasing need for certain healthcare specialists [46]. In addition, both availability and accessibility of facilities for physical activity and community socialization play a central role [47]. Apart from OD derived from official statistics, geodata has become a valuable resource for mapping healthcare supply realities at different spatial levels.

### Physical environment

The physical environment has a major impact on health, particularly on a small-scale level. This refers to the mental, physical and social components [48]. The land use mix, i.e. the proportion of open spaces, traffic areas, buildings and important supply infrastructure, is a key factor in this context. Regional and local cadastral offices mostly provide information on public green spaces as OD. Moreover, openly accessible indicators such as land coverage, building density and soil sealing can reflect these aspects, while providing insights into the ecological situation and climate-relevant stresses. For instance, a high degree of sealing possibly harbors a local potential for overheating and thus the risk of heat stress. Last but not least, extensive industrial areas point towards an increased production of exhaust gases [49]. In contrast, low-density areas with less soil sealing are often characterized by a high proportion of green space, which serves as a filter for pollutants and a source of oxygen, reducing air temperature and humidity. When it comes to open, green and recreational spaces, there are several OD resources (Attachment 1, Table 3).

### Climate and environment

Environmental hazards are physical, chemical and biological factors affecting individuals externally. Of these, air pollution is considered to contribute to diseases with the greatest attributable burden of disease [50]. Commonly established indicators for measuring the exposure include the emission of the substances sulfur dioxide (SO2), nitrogen oxides (NOX), ammonia (NH3) and particulate matter. Based on the Federal Emmission Control Act (BImSchG), measuring stations are maintained to determine and monitor air pollution levels, especially in regions with high traffic volumes (including large cities and metropolitan areas). Information on the spatial dispersion of such pollutant concentrations is partly provided through geostatistical interpolations.

However, with proximity to traffic being a relevant criterion, total annual emissions (in metric tons) in predefined administrative units are of restricted explanatory value. Thus, there is limited applicability for deriving area-wide, small-scale exposures (e.g. daily concentrations for fine particulate matter). This also applies to other data collected only at specific measuring points, such as meteorological data of the DWD. Direct environmental influences also appear as health consequences due to climate or environmental noise. Noise maps for road traffic are usually available for large cities with more than 100,000 inhabitants, in some cases as comprehensive model calculations and with data from noise appraisals. For smaller cities and communities, however, the situation is more varied. Likewise, climate data (and climate function maps) are available in some cases, especially for small-scale areas. Soil and water quality data (factors affecting health indirectly) is largely available at most scales.

### Spatial level and data availability

Although there is political and public interest in implementing health-promoting policies, especially on a municipal level, problems arise especially when it comes to small-scale analyses. For data-protection reasons, raw data is not provided for the smallest geographical units but processed or aggregated for specific purposes instead.

Moreover, different administrative boundaries and aggregations make it difficult to compare and link data with attribute data. Besides said limited availability and reduced ability for regionalization, data usage is mainly hampered by federal structures. For instance, central portals such as *Gov.Data* can only host municipal datasets if the associated federal states (FS) support the portal. To date, however, only 12 FS have joined the agreement. Instead, some federal states have launched their own data portals. As a result, the amount of data, access to it and the legal framework are heterogeneous and, in some cases, do not come with any legal regulations based on the Freedom of Information Act (IFG). Even the municipal GBE is focused on a state- or region-specific perspective and does not exhibit a common basic standard.

**Figure 2:**
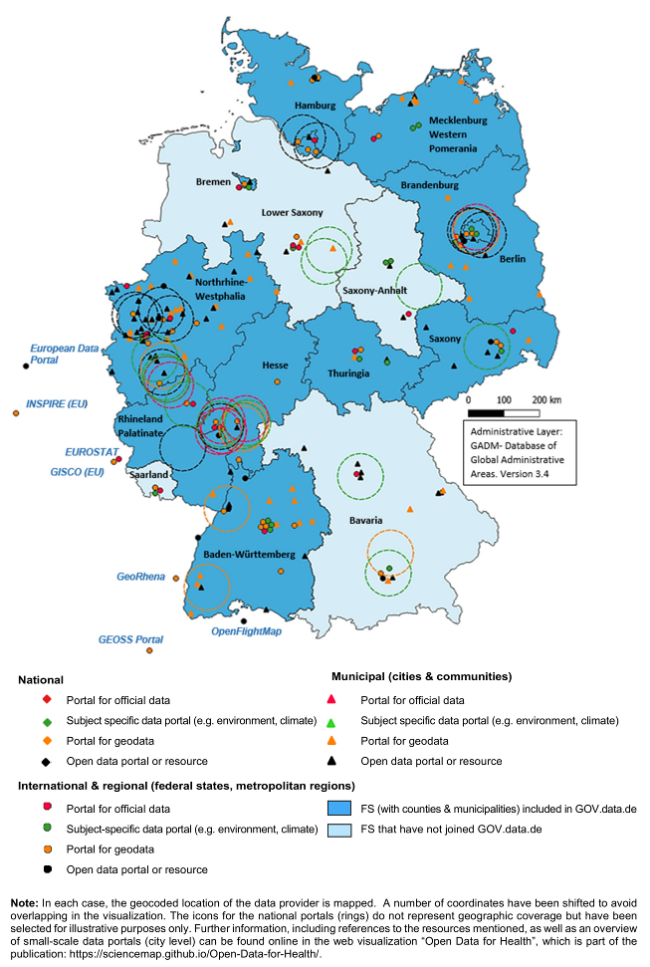
Availability of open data by spatial scale and data source (up to April 2021)

Despite the slight increase in the number of municipalities publishing OD (especially in North Rhine-Westphalia, Hamburg and Berlin), the provision is currently still very selective or, in some regions, almost non-existent (Figure 2 (Fig. 2)). At different spatial scales, some parties provide extensive OD, while others offer thematically non-comparable OD or no OD at all (especially in low-finance local areas). In 2021, out of more than 11,000 municipalities in Germany, only about 146 made any data available at all, some of them with less than 10 datasets [51]. Accordingly, the number is further reduced for the context discussed here. Attachment 1, Table 4 provides an overview of data resources, data types and licenses by different administrative level, which could have been identified based on this current research.

## Discussion

Availability, interdisciplinary use and linkage of data are essential for the development and monitoring of health strategies and are in line with the understanding of *health in all* policies. Therefore, public health research seeks to make ‘data for action’ more visible, accessible and disseminable [52] to identify the need for political action.

In the light of ongoing digitization and the resulting increase in data quantity, quality and use, extensive opportunities have opened up for analyzing associations between space and health, ranging from regional to international levels.

In this context, open access to data is a necessary requirement. The data resources highlighted above demonstrate that much data, in particular open geodata, is available in principle, but also that information on how to access it is crucial. Furthermore, in many cases it remains unclear what data is actually available as OD. It is difficult to determine the current supply status of OD, as there is no central or official body to collect this information.

Due to some limitations, the increasing number of different portals provides, at best, an entry point for research. Freedom of information and data-protection laws, which are either specific to each portal or lacking, represent major complicating factors.

Alternate access via search-engine queries, however, first requires indexing of the data, e.g. based on technical openness and accompanied by metadata [12]. The technical, legal and content-related heterogeneity of the offerings discussed above as well as regulatory conditions still hinder the systematic use and thus full exploitation of the potential offered by these resources. For instance, a variety of data is accessible in principle but cannot be retrieved in machine-readable, open form.

Furthermore, both quantity and quality of the available data vary. In particular, small-scale and comprehensive availability is patchy and highly heterogeneous. Despite some initiatives such as INSPIRE, finding and accessing geospatial data also remains somewhat challenging. Since health-related geographic data is relatively scarce [53], surveillance by the Robert Koch Institute, for example, hardly takes any advantage of INSPIRE so far [25].

Data openness also covers aspects of participation and continued (re)usage. Although administrative data providers are encouraged to share raw data in accordance with the E-Government Act, the decision on how to use the data still remains with the authorities themselves. In addition, the absence of an explicit license prevents further use in many cases. The Geodata Access Act (GeoZG) also calls for no-cost data sharing but imposes restricted reuse that often incurs a fee. Thus, there is still a mixed landscape in terms of fees and (licensing) policies for geodata, which makes it difficult to merge data in a comprehensive manner. On the one hand, Open Citizen projects such as OSM are a valuable source in this context, not just in terms of data volume. On the other hand, this data exhibits certain disadvantages in terms of completeness, quality, topicality and validity. In contrast, official geodata is often available for administrative levels but not applicable to scientific analyses. As an example, small-scale epidemiology data is frequently provided on a postal code level, which overlaps inconsistently with administrative units. Other challenges of administrative geodata affecting spatial analyses and calling for alternative approaches are territorial reforms, data aggregations, scaling and zoning effects. In some cases, even a change of indicators may become necessary for area-wide or cross-level analyses for availability reasons.

## Outlook

For various reasons, it is currently not possible to give an exhaustive overview of OD offerings. Given that such overviews are only able to represent a snapshot in a dynamically changing context, it would be useful to expand them on an ongoing basis. A desirable feature might be portals/overviews using software to import relevant, ideally standardized, data catalogs from decentralized resources. Metadata (including technical and legal conditions) is a key basis and requires the use of (meta)data catalogs as well as unique identifiers for data records supplied by all data providers.

The provision of OD is a joint process involving several micro and macro levels (state, federal states, counties, municipalities, institutions). It also implies that, besides urgently needed harmonization of legal aspects, general processes must be redesigned starting already in the context of (official) data collection and research to initially give all data the option of openness and machine readability. In particular, the institutional and administrative bodies responsible for quality control, data management and data protection should be integrated into such strategies and processes at an early stage.

Regarding the further use of research data in the course of FAIRification, discipline-specific efforts to achieve common standards, e.g. ontologies and vocabularies, would have to be pushed forward to optimize the findability and indexing of data and metadata sets in both thematic databases and general search engines. To improve the performance of the latter, Google, Yahoo, Yandex and Bing have already developed the *schema.org* metadata standard, which helps data-producing and data-providing parties structure data according to uniform vocabulary. The increased integration of Linked Data standards with the delivery process can also significantly improve data findability, accessibility and comparability in the future.

To ensure the potential further use of data, (harmonized) legal foundations are required on the level of the federal states and municipalities. Orientation for OGD on the municipal level is currently provided by the model data catalog, which lists types and sources of OGD [6].

Concerning OGD, the draft law dated February 2021 on the *Second Open Data Act and Data Utilization Act* (Section 12a EGovG) [54] could have been an important milestone. Although its passing (of June 24, 2021) obligates authorities of the intermediate federal administration to offer unprocessed, machine-readable data to the public, the claim upon this provision (also for non-protected, personal data) is still restricted. Data from private companies (including public transport) and publicly funded research data, which are newly included in this law, are also partly affected by extensive, non-OD-conform restrictions. For instance, the offering parties may charge fees for their data and decide on licensing (and thus on further use). Consequently, one overarching future goal must be to create general incentives for OD – not least in terms of financial and human resources. Within the public health research landscape, the growing potential of data sharing, despite the (initial) costs of FAIRification and the opening of data in line with OD principles, should be acknowledged, actively requested and used.

## Abbreviations

- BBSR: Bundesinstitut für Bau-, Stadt- und Raumforschung (Federal Institute for Building, Urban and Spatial Research)
- BImSchG: Bundesimmissionsschutzgesetz (Federal Emmission Control Act)
- BKA: Bundeskriminalamt (Federal Criminal Police Office)
- BKG: Bundesamt für Kartographie und Geodäsie (Federal Agency for Cartography and Geodesy)
- BMVI: Bundesministerium für Verkehr und digitale Infrastruktur (Federal Ministry of Transport and Digital Infrastructure)
- CC: Creative Commons
- DKG: Deutsche Krankenhausgesellschaft (German Hospital Association)
- DWD: Deutscher Wetterdienst (Germany’s National Meteorological Service)
- FOIA: Freedom of Information Act
- FS: Federal States
- GBE: Gesundheitsberichterstattung (Federal Health Reporting)
- GDI-DE: Infrastructure for Spatial Information in Germany
- GeoZG: Geodatenzugangsgesetz (Geodata Access Act)
- GISD: German Index of Socioeconomic Deprivation
- INKAR: Indikatoren und Karten zur Raum- und Stadtentwicklung (Indicators and maps for spatial and urban development)
- INSPIRE: Infrastructure for Spatial Information in Europe
- OD: open data
- ODbL: Open Database License
- ODC: Open Data Commons
- ÖPNV: Öffentlicher Personennahverkehr (public transport)
- OGD: open government data
- OSM: OpenStreetMap
- PDDL: Public Domain Dedication and License
- PKS: polizeiliche Kriminalitätsstatistik (federal-level police crime statistics)
- SUF: Scientific Use Files
- UBE: Umweltberichterstattung (environmental reporting)
- WHO: World Health Organization

## Links to the resources mentioned within the text

- CODE-DE2: https://code-de.org/en/
- Data portal of Deutsche Bahn AG: https://data.deutschebahn.com/
- ESRI Open Data: https://opendata-esri-de.opendata.arcgis.com/
- Google Maps: https://about.google/brand-resource-center/products-and-services/geo-guidelines/
- Hospital Locations: https://krankenhausstandorte.de
- INKAR: https://www.inkar.de/
- mCLOUD: https://www.mcloud.de/
- mdm Portal: https://www.mdm-portal.de/
- National social welfare statistics: https://www.statistikportal.de/de/sbe
- NVBW (Local transport company Baden-Württemberg) Open Data: https://www.nvbw.de/open-data
- OD portal of the German Weather Service: https://www.dwd.de/EN/ourservices/_functions/search/search_Formular.html
- OpenDataAtlas: http://opendata.tursics.de/
- Opendata-City-Census: http://de-city.census.okfn.org
- Open Data for Health Map: https://sciencemap.github.io/Open-Data-for-Health/
- Open Data Platform ÖPNV: https://www.opendata-oepnv.de
- OpenSenseMap: https://OpenSenseMap.org
- OpenStreetMaps: https://www.openstreetmap.org
- Regional social welfare statistics (examples): https://statistikportal.thueringen.de/thonsa/SSDstart.php and http://www.gsi-berlin.info/
- Registry of Research Data Repositories: http://re3data.org
- Sensor Community: https://sensor.community/en/

## Data

Data for this article are available from the Dryad Repository: https://doi.org/10.5061/dryad.djh9w0w1f [55].

## Notes

### Acknowledgments

The authors would like to thank NFDI4Health for the inspiration and knowledge sharing. This was of great value for the whole work process.

### Competing interests

The authors declare that they have no competing interests.

## Supplementary Material

Availability of data in the context of space and health, Overview of data access

## Figures and Tables

**Table 1:**
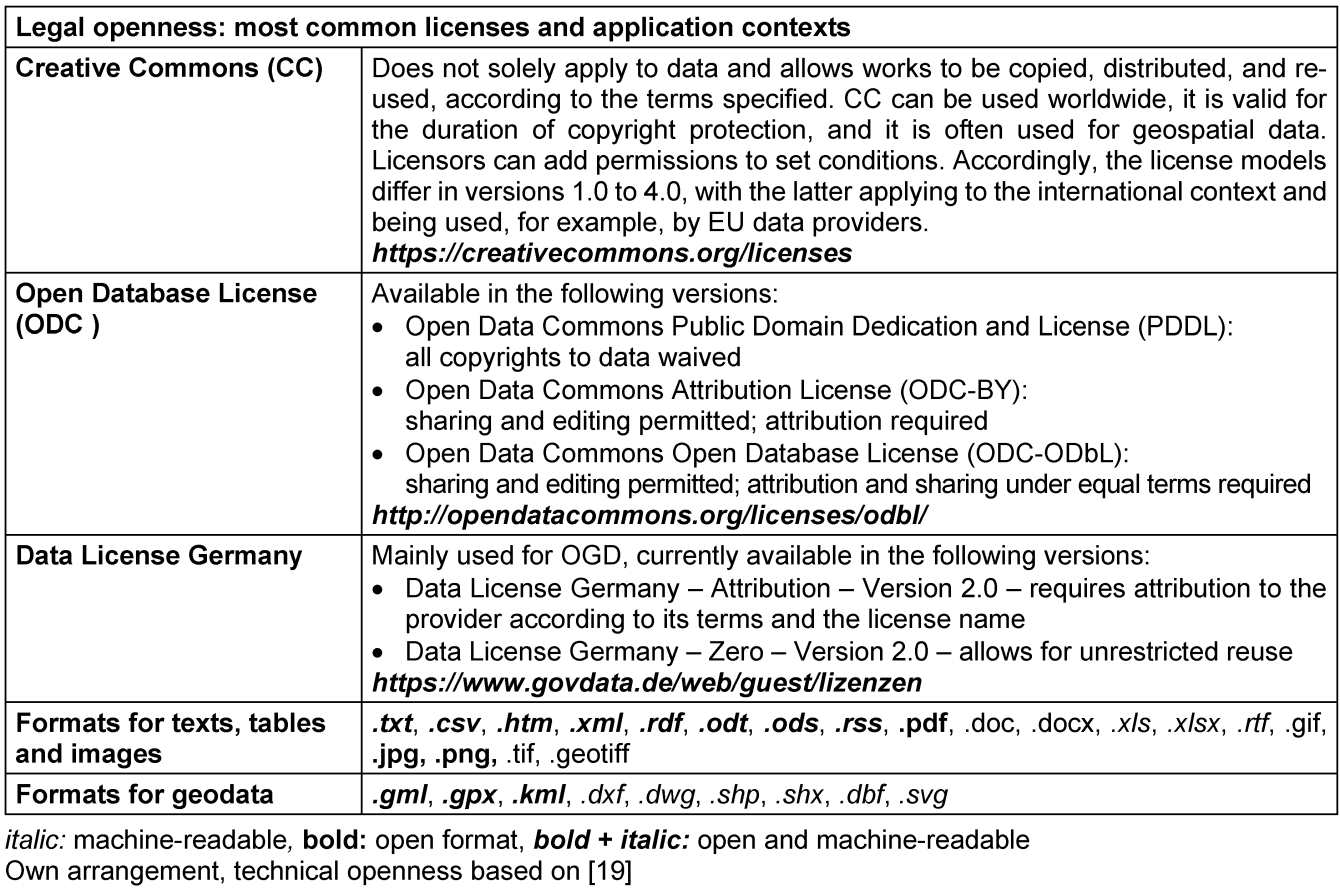
Legal and technical openness

**Table 2:**
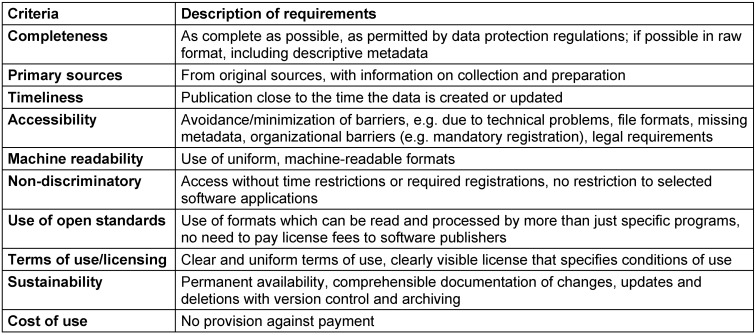
Open data criteria [56]
