# Supplementary material for: Availability of open data for spatial public health research
Source: Ger Med Sci. 2022 Mar 4;20:Doc01. doi: 10.3205/000303 (PMC9006316; doi:10.3205/000303)
Supplement: Availability of data in the context of space and health, Overview of data access [file GMS-20-01-s-001.pdf]

Table 3: Availability of data in the context of space and health by spatial scale starting from county level

Dimension/ Spatial relevance	Indicator	Counties/metropolitan regions		Local level (municipalities)		Micro level (city, districts, neighborhoods)	
		**Availability**	**Sources/Examples**	**Availability**	**Sources/ Examples**	**Availability**	**Sources/Examples**
**Social environment factors**<br><br>*Sociodemographic and socioeconomic factors, degree of urbanization, social infrastructure, community well-being, and safety*	Population density, housing density	Within portals of official statistics on European, national and state level, in (open) data portals of certain districts, within state, superordinate collective portals (e.g. metadata or OD catalogs), in listings of citizens' initiatives – partly available combined with geo-information	DESTATIS, statistikportal.de, statistical offices of the federal states, INKAR, ZEFIR, partly EUROSTAT, GISCO, Gov.data.de, metaver.de (for 7 federal states), INSPIRE geoportal, Geoportal.de, mCLOUD, offenedaten.de, census2011.de, a few OD portals of federal states/counties, GBE	Same as county level + municipal OGD or OD portals		Selected availability only	Partly within city statistics, city OGD or city OD portals, Open Data city census
	Average age of population, proportion of 65–79-year-olds per population, old-age dependency ratio, net migration, proportion of foreigners, population development						
	Tax revenues per inhabitant, municipal debt, GDP						
	Unemployment, employment rate, SGB II rate						
	Education						
	Gross wages & salaries, dispos. HH income						
	Public libraries	Official data (some of them not machine-readable), CSD	State library catalogs/statistics, OSM	Same as district level		CSD	OSM
	Cultural facilities (cinemas, theaters, museums, clubs, etc.)	Difficult to find in official statistics (hardly any open formats), CSD	Federal state statistics portals, OSM	Same as district level		CSD	OSM
	Broadband Internet	Partly official statistics, OGD, OS	INKAR, Broadband Atlas (BMVI), Thünen Land Atlas	Same as district level		Selected availability only	Broadband Atlas (BMVI), Thünen Land Atlas
	Living space per person	Official statistics, OS	Partly state-specific statistics portals, ZEFIR (SUF-File)	Same as district level		Selected availability only	Partly in city OD or statistics portal

Attachment 1 to: Peters M, Zeeb H. Availability of open data for spatial public health research. GMS Ger Med Sci. 2022;20:Doc01. DOI: 10.3205/000303, URN: urn:nbn:de:0183-0003030

	Injured/killed in road traffic	Official statistics, OS	Federal State Statistical Offices, ZEFIR (SUF)	Partly official statistics (Federal State Offices, municipal statistics)		Selected availability only	Partly in city portals
	Availability of footpaths & cycle paths	CSD	OSM	CSD	OSM	OS	OSM
	Crime	OGD (available only very heterogeneously, in part in non-machine-readable formats. Formats: since 2020 as .xls, until 2019 as PDF), often only upon request)	BKA	Same as district level	Some municipal OD portals, BKA	Partly in city OD portals, partly OGD for cities with ≥100,000 p.e.	Partly BKA, partly city-based, e.g. kriminalitaetsatlas.berlin.de
**Availability, access to, and accessibility of health-related resources**	Public transport infrastructure/public transport stops	Open company data, CSD	INKAR, OSM, opendata-oepnv.de, mCLOUD, Open Data DB	Official statistics, open company data, CSD	INKAR, OSM, opendata-oepnv.de, Open Data DB	Open company data, CSD	OSM, opendata-oepnv.de, Open Data DB
Medical care, green and blue spaces, sports facilities	Car occupancy	Official data/OGD	Federal State Statistical Offices, govdata.de	Partly in official statistics	Federal State Statistical Offices	Selected availability only	Partly in city portals
	Travel times to central and regional centers	Official data, CSD	INKAR, OSM, INSPIRE, geoportal.de	Same as district level		CSD[2]	OSM
	Accessibility of IC/ICE train stations, supermarkets, physicians, pharmacies						
	P.E. per physician						
	Places in care facilities						
	Number & accessibility of hospitals providing general care	Official statistics, CSD	INKAR, DESTATIS (Krankenhausatlas, OSM)	Same as district level		Same as district level	
	Number of hospital beds per 1000 inhabitants	Official statistics	INKAR, Federal State Statistical Offices	Same as district level, OS, (Thünen Landatlas)		OS	Thünen Landatlas

**Physical environment**	Public green/aquatic/ recreational spaces, commercial/industrial/ traffic areas[1]	Official statistics, OGD, CSD	ZEFIR, INKAR, OSM, BfK, state portals for geodata, INSPIRE, govdata.de, BKG, geportal.de	Same as district level, partly on Municipal Portals		Partly on city statistics or OD portals, OS	In city portals, OSM
**Climate and environment**<br><br>*Air pollution, climate exposure*	Thermal exposure (heat, cold)	OGD, OGEOD (as GRIDS)	OD des DWD	Same as district level, partly on Municipal OD Portals		Same as district level, partly data on city OD portals	
	UV radiation	OGEOD (as GRIDS, GJSON, .csv)	DWD, BfS-Geoportal	Same as district level, partly on Municipal OD Portals		Same as district level, partly data on city OD portals	
	Soil sealing	Official statistics, OGD, OD portals, CSD	INKAR, OSM, BfK, State portals for geodata	Same as district level		Partly in local portals, CSD	In city portals, OSM
	Particulate matter, ozone, CO2, nitrogen oxides	CSD, subject data on request at UBA, partly in state portals (often not machine-readable)	UBA, partly in federal state/county portals, luftdaten.info	Same as district level	UBA, partly in municipal portals, luftdaten.info	Same as district level	UBA, partly in city portals, luftdaten.info, www.codefor.de
	Noise	Available heterogeneously as OD/ OGEOD	Partly in federal state portals	Same as district level	Partly in municipal portals	Same as district level	Partly in city portals
	Drinking water quality	Only available on a small scale, mainly in non-open formats				With municipal water suppliers, in some cases information only available upon request	
	Radon	OGEOD als GJSON, .csv	BfS-Geoportal	Same as district level		Same as district level	

[1]Data available as proportion values or per 1000 p.e. km^2, [2]own calculations based on data needed/possible

BfS = Bundesamt für Strahlenschutz (Federal Office for Radiation Protection), BKA = Bundeskriminalamt (Federal Bureau of Investigation), BMVI = Bundesministerium für Verkehr und digitale Infrastruktur (Federal Ministry of Transport and Digital Infrastructure), CSD = Crowd-Sourced Data, DB = Deutsche Bahn, HH = Household, INKAR = Indikatoren und Karten zur Raum- und Stadtentwicklung (Indicators and maps of spatial and urban development), KBA = Kraftfahrzeugbundesamt (Federal Motor Transport Authority), OD = Open Data, OGD = Open Government Data, OGEOD = Open Geo Data, OSM = OpenStreetMaps, OS = Open Science, SUF = Scientific Use File, ZEFIR = Zentrum für interdisziplinäre Regionalforschung (Center for Interdisciplinary Regional Research)

Further information, including links to the resources mentioned, as well as an overview of small-scale portals (city level) can be found in the web visualization that accompanies the publication: "Open Data for Health Map", https://sciencemap.github.io/Open-Data-for-Health.

Table 4: Overview of data access, data types and licenses by administrative level (last updated April 2021)

National and international portals				Portals of the federal states and metropolitan regions			
**Level**	**Title/description**	**Data type**	**License**	**State/region**	**Title/description**	**Data type**	**License**
International	Open Data Portal of the EU		CC BY 4.0	Brandenburg	GDI Brandenburg		Per resource
International	European Data Portal		Per resource	Brandenburg	Landscape & Environment Information		Per resource
International	EUROSTAT		CC BY 4.0	Brandenburg	Open Data Brandenburg		DL-DE->BY-2.0
International	GISCO		CC BY 4.0	Brandenburg	Geobroker LGB		Per resource
International	INSIPRE Geoportal		Per resource	Brandenburg	Open Geodata Brandenburg		DL-DE->BY-2.0
International	OPENAIRE		CC BY 4.0	Bremen	GDI Bremen		Per resource
International	GEOSS Portal		Per resource	Bremen	Environment Information Bremen		Per resource
International	OpenFlightMap		OFMA General Users	Bremen	Demographic Monitoring		Per resource
National	BKG – Open Data		DL-DE->BY-2.0	Germany	Atlas Regional Statistics		DL-DE->BY-2.0
National	GDI-DE – Geodata Germany		Per resource	Hamburg	GDI Hamburg		DL-DE->BY-2.0
National	DESTATIS		DL-DE->BY-2.0	Hamburg	Geodataportal Hamburg		Per resource
National	Deutsche Bahn Open Data		CC BY 4.0	Hamburg, SHS	Statistical Office Hamburg & SH		DL-DE->BY-2.0
National	Esri Germany Open Data		Per resource	Hesse	GDI Hessen		DL-DE->BY-2.0
National	Citizen Project Open Data		Per resource	Hesse	Statistical Office Hesse		DL-DE->BY-2.0
National	Portal for open government data		Per resource	MR Hamburg	Geoportal MR Hamburg		Per resource
National	GovData[1]		Per resource	MR Rhein-Neckar	Open Data MR Rhein-Neckar		Per resource
National	mCLOUD of BMVI		Per resource	MV	GDI MV		Per resource
National	Open Data Portal Municipalities		Per resource	MV	Statistical Office MV		DL-DE->BY-2.0
National	IÖR-Monitor		Per resource	MV	Map Portal Environment MV		Per resource
National	Metaver – Metadata Network		Per resource	MV	Environmental Information MV		Per resource
National	OpenStreetMap Germany		OdbL	Lower Rhine	Geoportal Lower Rhine		Per resource
National	Federal Environmental Agency		Per resource	Lower Saxony	GDI Lower Saxony		Per resource
National	Climate Data Center Portal		Per resource	Lower Saxony	Lower Saxony Environmental Portal		Per resource
National	German Weather Service		Per resource	Lower Saxony	Statistical Office Lower Saxony		DL-DE->BY-2.0
National	Statistics portal		DL-DE->BY-2.0	MR Northeast	Regional Monitoring Lower Saxony		Per resource
National	Zensus 2011		DL-DE->BY-2.0	MR Northwest	Regional Monitoring MR Northwest		DL-DE->BY-2.0
National	Open Data ÖPNV		Per resource	NRW	GDI NRW		Per resource

National	Data download page of the BBSR					DL-DE->BY-2.0	NRW	Open.NRW					Per resource
National	Germany Atlas interactive 2020					DL-DE->BY-2.0	NRW	NRW Local environmental data					Per resource
National	Data compilation of GBE					DL-DE->BY-2.0	NRW	OpenGeodata.NRW					Per resource
National	Spatial monitoring indicators					Per resource	NRW	Open Data Portal					DL-DE->BY-2.0
National	Regional Database Germany					DL-DE->BY-2.0	NRW	Statistical Office NRW					DL-DE->BY-2.0
National	Federal Office for Radiation Protection					Per resource	NRW	GBE – Data portal at district level					Per resource
National	Postal code areas					ODbL	NRW	Ministry of School & Education					DL-DE->BY-2.0
National	INSPIRE Germany					Per resource	Upper Rhine region	GeoRhena					Per resource
National	Geofabrik GmbH					ODbL 1.0	East Württemberg	Regional Association					Per resource
National	Atlas of Accidents					DL-DE->BY-2.0	Stuttgart region	RegioRISS Stuttgart					Per resource
National	ZEFIR					Own regulations	RP	GDI Rhineland Palatinate					Per resource
National	Thünen Atlas					Per resource	RP	Open Government Data Portal RP					Per resource
National	Care Atlas Germany					Per resource	RP	Statistical Office RP					DL-DE->BY-2.0
National	CODE-DE					Own license	RP	Environmental portal RP					Per resource
National	Data-Portal BMBF					DL-DE->BY-2.0	Ruhr region	Regional Association Ruhr					Per resource
National	Federal Agency for Nature Conservation					Per resource	Ruhr region	Open Data Portal Ruhr					Per resource
National	wasser.de					Per resource	Ruhr region	Geodata Portal					Per resource
National	Federal Institute of Hydrology					Per resource	Saarland	GDI Saarland					Per resource
National	German Library Statistics					Own regulations	Saarland	Statistical Office Saarland					DL-DE->BY-2.0
National	Police crime statistics					DL-DE->BY-2.0	Saarland	Environmental portal Saarland					Per resource
National	ESRI Crime					DL-DE->BY-2.0	Saarland	GDI Saarland					Per resource
**Portals of the federal states and metropolitan regions**							Saxony	GDI Saxony					Per resource
BA-WÜ	GDI Baden-Württemberg					DL-DE->BY-2.0	Saxony	Open Data Saxony					DL-DE->BY-2.0
BA-WÜ	Geoportal Spatial Planning					Per resource	Saxony	Statistical Office					DL-DE->BY-2.0
BA-WÜ	State Statistical Office					DL-DE->BY-2.0	Saxony	Environmental portal Saxony					Per resource
BA-WÜ	Data and map service of the LUBW					Per resource	Saxony	Open Geodata Saxony					DL-DE->BY-2.0
BA-WÜ	Environmental portal BA-WÜ					Per resource	Saxony-Anhalt	State geodesy Saxony-Anhalt					DL-DE->BY-2.0
BA-WÜ	State Office for Geoinformation					Per resource	Saxony-Anhalt	Statistical Office					DL-DE->BY-2.0
BA-WÜ	GDI Baden-Württemberg					Per resource	Saxony-Anhalt	Environmental Information Network					Per resource
BA-WÜ	Ministry of Rural Areas					DL-DE->BY-2.0	SHS	GDI SHS					Per resource
BA-WÜ	KITopenData					Per resource	SHS	Environmental Portal SHS					Per resource

Bavaria	GDI Bavaria					Per resource	SHS	Digital Atlas North					Per resource
Bavaria	Open Data Bavaria					Per resource	SHS	Open Data Portal SHS					Per resource
Bavaria	Bavarian State Office for Statistics					DL-DE->BY-2.0	Schwelm	Geodata portal Ennepe-Ruhr-Kreis					Per resource
Bavaria	Environmental Information Systems Bavaria					Per resource	South Hesse	GDI South Hesse					Per resource
Berlin	GDI Berlin					Per resource	Southwestphalia	GDI Southwestphalia					Per resource
Berlin	Environmental Portal Berlin (ISU)					Per resource	Thuringia	GDI Thuringia					Per resource
Berlin	Crime Atlas Berlin					DL-DE->BY-2.0	Thuringia	Statistical Office					Per resource
Vogelsberg	Geoportal Vogelsbergkreis					Per resource	Thuringia	Institute for Environment & Geology					Per resource
Rhine-Main	Geoportal of the regional association					Per resource	Thuringia	Environmental Portal Thuringia					Per resource

	Open data on the social environment (social condition, sociodemographic and socioeconomic factors)
	Open (geo)data on availability, access, and accessibility of health-related resources
	Open (geo)data on the physical environment
	Open subject/geo-data on climate and environment

[1]Only for the federal states of Brandenburg, Bremen, Hamburg, Mecklenburg-Western Pomerania, Saarland, Saxony, Saxony-Anhalt
BA-WÜ= Baden Württemberg, DWD = Deutscher Wetterdienst (German weather service), GDI = Geodateninfrastruktur (Spatial Data Infrastructure), MR = Metropolitan Region, MV = Mecklenburg-Western Pomerania, NRW = North Rhine-Westphalia, RP = Rhineland Palatinate, SHS = Schleswig-Holstein

Further information, including links to the resources mentioned, as well as an overview of small-scale portals (city level) can be found in the web visualization that accompanies the publication: „Open Data for Health Map“, https://sciencemap.github.io/Open-Data-for-Health.
